# Supplementary figures and images for: driveR: a novel method for prioritizing cancer driver genes using somatic genomics data
Source: BMC Bioinformatics. 2021 May 24;22:263. doi: 10.1186/s12859-021-04203-7 (PMC8142487; doi:10.1186/s12859-021-04203-7)

**A**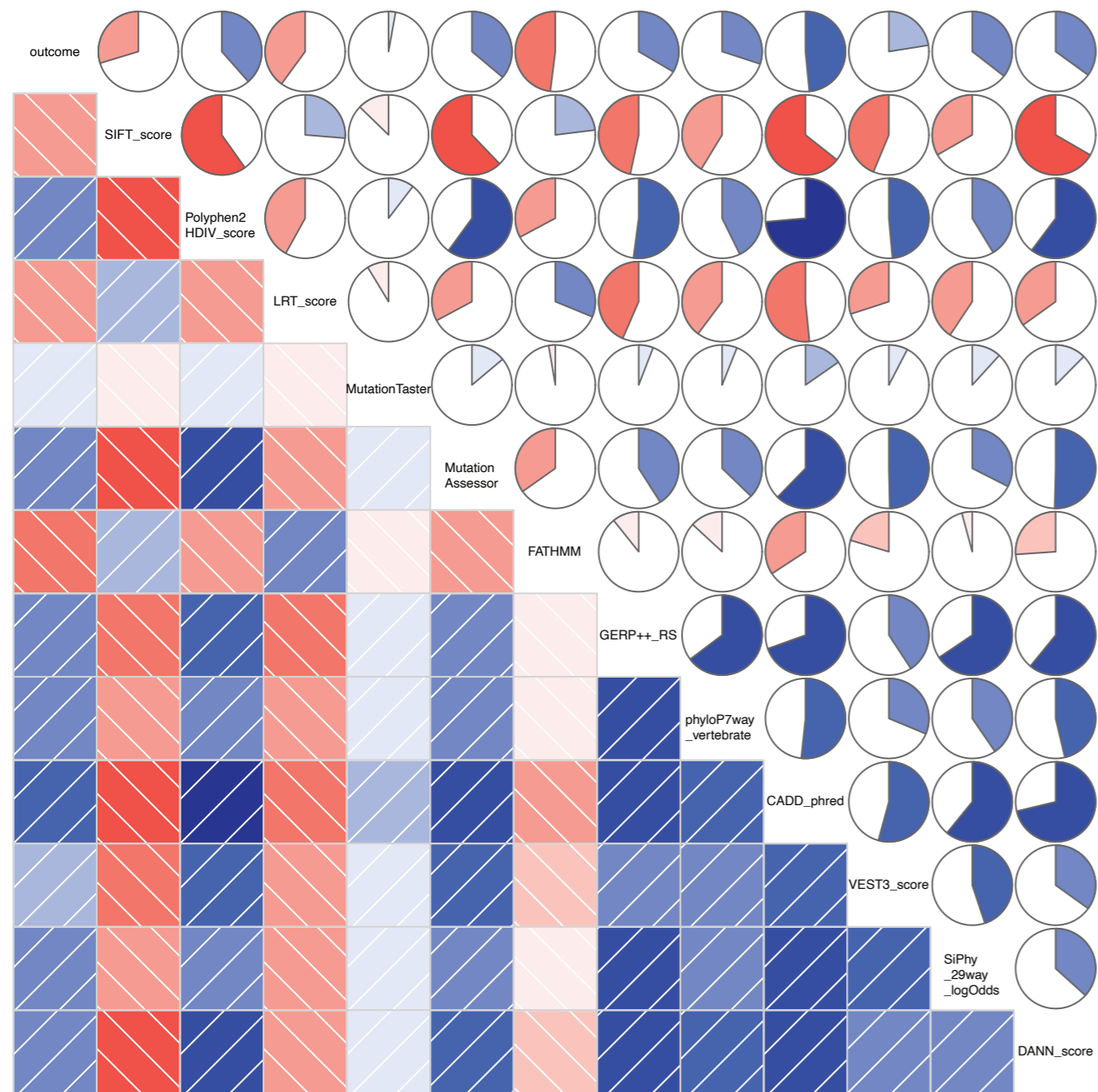**B**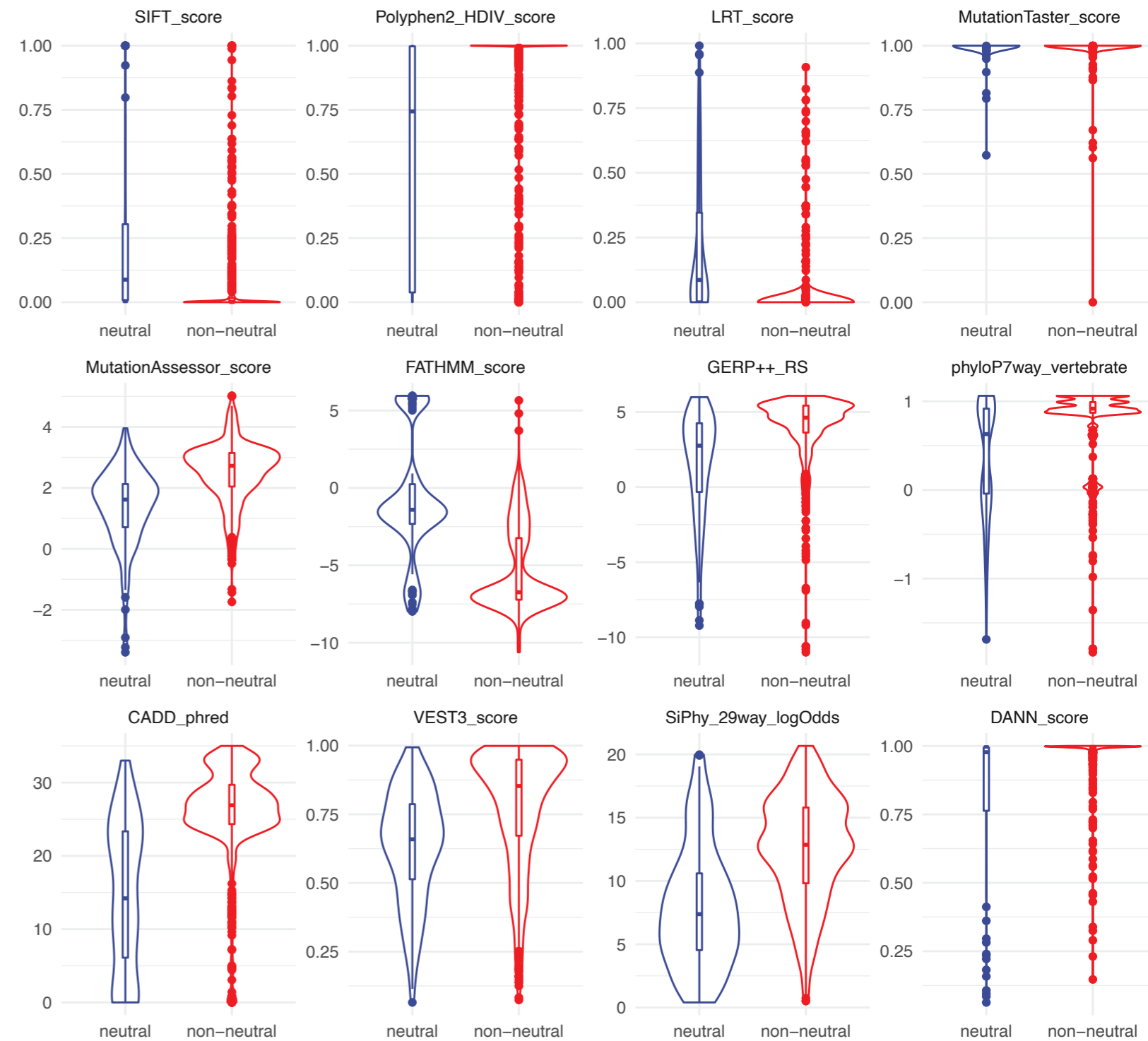

Supplement: Supplementary file 1 — Additional file 1: Figure S1. The overall analysis of individual variant impact predictors. (A) Correlogram displaying correlations between the outcome and individual variant impact predictors. (B) Violin plots displaying the distributions of scores of individual variant impact predictors in driver and non-driver variants. [file 12859_2021_4203_MOESM1_ESM.pdf]

**A**

## Performance on the training dataset

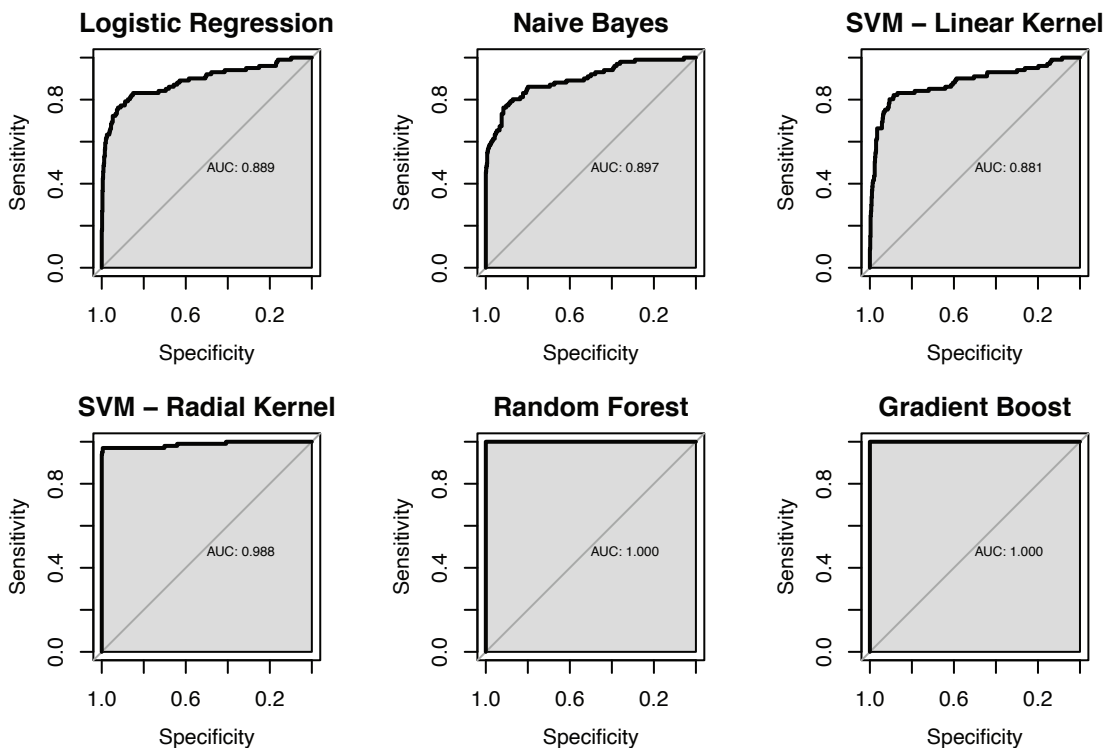**B**

## Performance on the test dataset

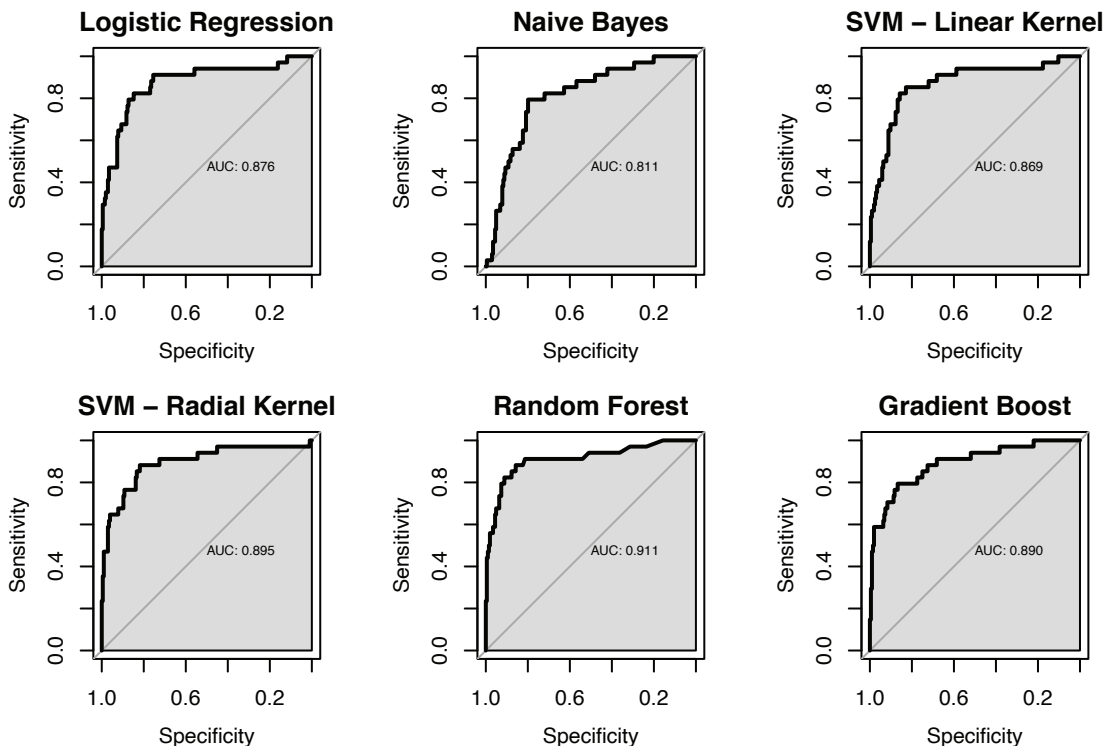

Supplement: Supplementary file 2 — Additional file 2: Figure S2. Performance of different coding variant impact metapredictor models in the training (A) and test (B) datasets. [file 12859_2021_4203_MOESM2_ESM.pdf]

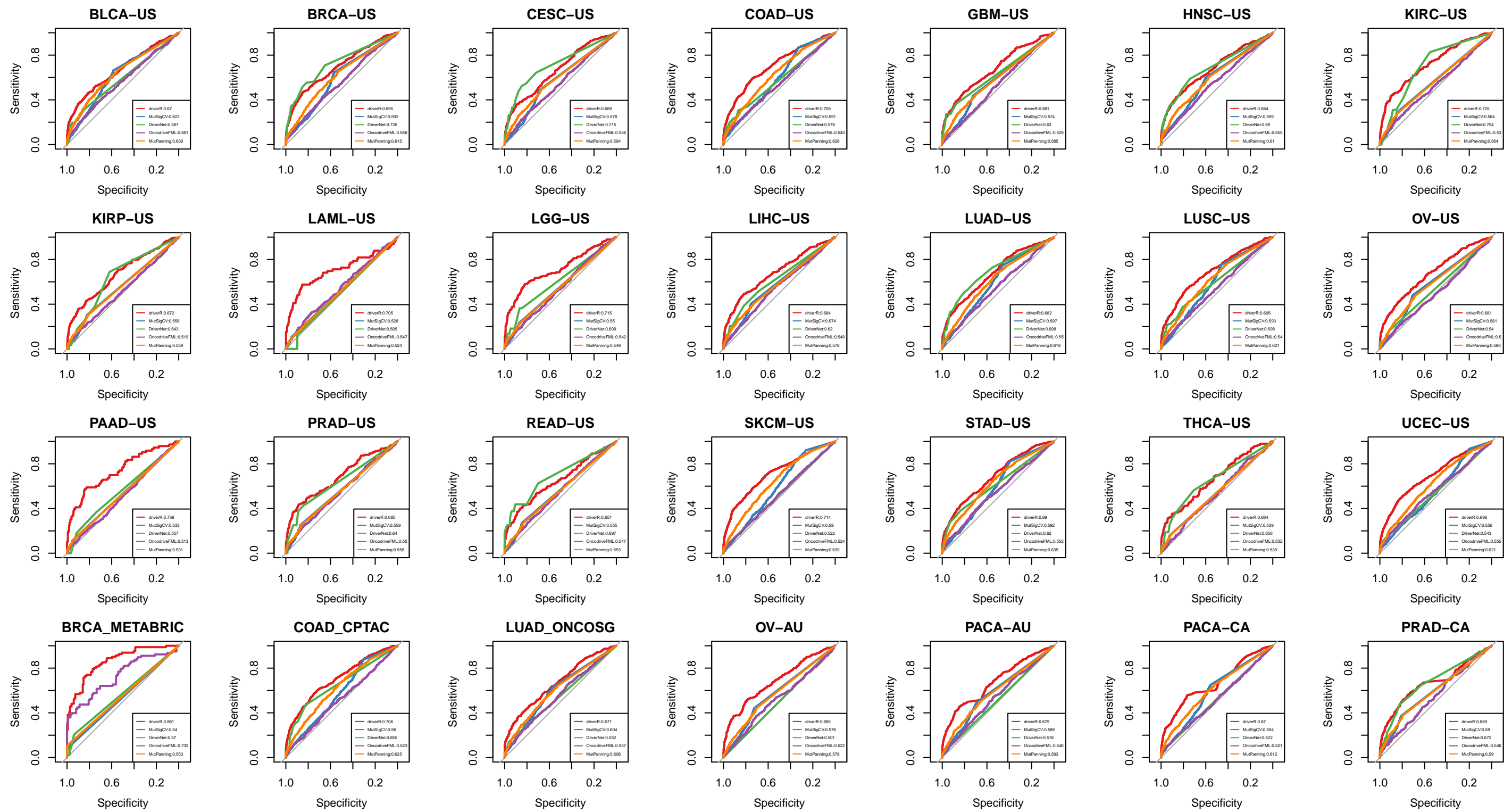

Supplement: Supplementary file 3 — Additional file 3: Figure S3. Comparison of performance of driveR with batch analysis approaches per test dataset. ROC curves for assessing the performance of each approach per each test dataset. The bottom-right legends display AUC per each approach. [file 12859_2021_4203_MOESM3_ESM.pdf]

All Test Datasets – AUC Distributions

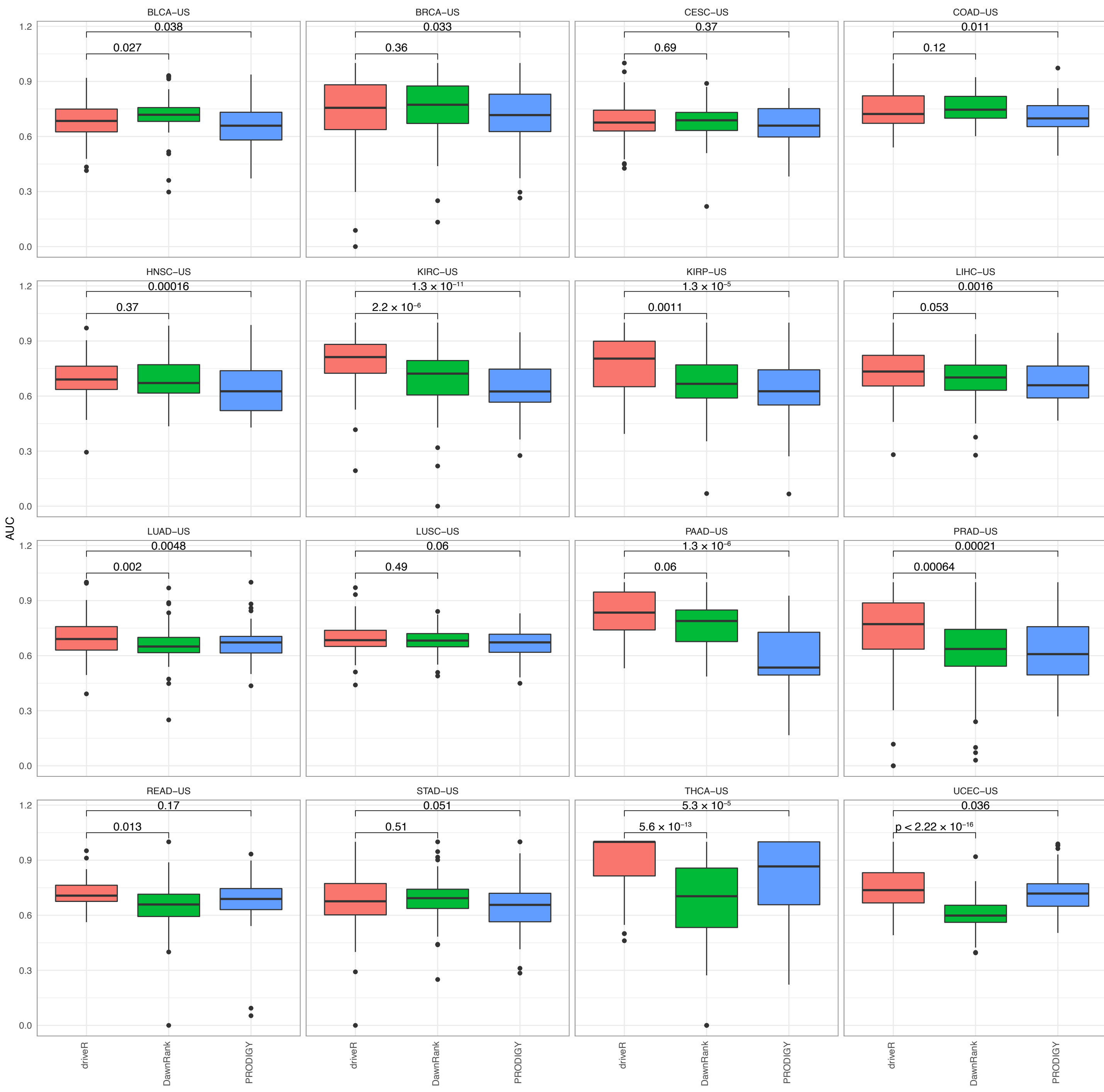

Supplement: Supplementary file 4 — Additional file 4: Figure S4. Comparison of performance of driveR with personalized analysis approaches per test dataset. Boxplots displaying the distributions of AUC values of each approach across all patients per test dataset. The bracket display p values per each comparison. [file 12859_2021_4203_MOESM4_ESM.pdf]
